# Non-Invasive Quantification of Fraction Flow Reserve Based on Steady-State Geometric Multiscale Models

**DOI:** 10.3389/fphys.2022.881826

**Published:** 2022-04-12

**Authors:** Jincheng Liu, Xue Wang, Bao Li, Suqin Huang, Hao Sun, Liyuan Zhang, Yutong Sun, Zhuo Liu, Jian Liu, Lihua Wang, Xi Zhao, Wenxin Wang, Mingzi Zhang, Youjun Liu

**Affiliations:** ^1^ Faculty of Environment and Life, Beijing University of Technology, Beijing, China; ^2^ Cardiovascular Department, Peking University People’s Hospital, Beijing, China; ^3^ Radiology Department, The Second Affiliated Hospital, Zhejiang University School of Medicine, Zhejiang, China; ^4^ Clinical and Technical Support, Philips Healthcare, Shanghai, China; ^5^ Depart of Biomedical Sciences, Macquarie Medical School, Macquarie University, Sydney, NSW, Australia

**Keywords:** coronary heart disease, fractional flow reserve, geometric multiscale, fast calculation of FFR, non-invasive diagnosis of myocardial ischemia

## Abstract

**Background:** The underuse of invasive fraction flow reserve (FFR) in clinical practice has motivated research towards its non-invasive prediction. The early attempts relied on solving the incompressible three-dimensional Navier–Stokes equations in segmented coronary arteries. However, transient boundary condition has a high resource intensity in terms of computational time. Herein, a method for calculating FFR based on steady-state geometric multiscale (FFR_SS_) is proposed.

**Methods:** A total of 154 moderately stenotic vessels (40–80% diameter stenosis) from 136 patients with stable angina were included in this study to validate the clinical diagnostic performance of FFR_SS_. The method was based on the coronary artery model segmented from the patient’s coronary CTA image. The average pressure was used as the boundary condition for the inlet, and the microcirculation resistance calculated by the coronary flow was used as the boundary condition for the outlet to calculate the patient-specific coronary hyperemia. Then, the flow velocity and pressure distribution and the FFRss of each coronary artery branch were calculated to evaluate the degree of myocardial ischemia caused by coronary stenosis. Also, the FFR_SS_ and FFR_CT_ of all patients were calculated, and the clinically measured FFR was used as the “gold standard” to verify the diagnostic performance of FFR_SS_ and to compare the correlation between FFR_SS_ and FFR_CT_.

**Results:** According to the FFR_SS_ calculation results of all patients, FFR_SS_ and FFR have a good correlation (r = 0.68, *p* < 0.001). Similarly, the correlation of FFR_SS_ and FFR_CT_ demonstrated an r of 0.75 (95%CI: 0.67–0.72) (*p* < 0.001). On receiver-operating characteristic analysis, the optimal FFR_SS_ cut point for FFR≤0.80 was 0.80 (AUC:0.85 [95% confidence interval: 0.79 to 0.90]; overall accuracy:88.3%). The overall sensitivity, specificity, PPV, and NPV for FFR_SS_ ≤0.80 versus FFR ≤0.80 was 68.18% (95% CI: 52.4–81.4), 93.64% (95% CI: 87.3–97.4), 82.9%, and 91.1%, respectively.

**Conclusion:** FFR_SS_ is a reliable diagnostic index for myocardial ischemia. This method was similar to the closed-loop geometric multiscale calculation of FFR accuracy but improved the calculation efficiency. It also improved the clinical applicability of the non-invasive computational FFR model, helped the clinicians diagnose myocardial ischemia, and guided percutaneous coronary intervention.

## Introduction

Over the last 10 years, fractional flow reserve (FFR) has become a reference standard for the invasive assessment of coronary artery disease. Its measurement assesses the functional severity of coronary artery stenoses and the need for coronary revascularization (Pijls et al., 1996; Pijls, 2013). FFR is calculated by dividing the distal coronary pressure (Pd) by the proximal coronary pressure (Pa) during maximal hyperemia (Pijls et al., 1996) and the diagnostic threshold is 0.80. FFR carries a Class 1a recommendation for guiding revascularization in angiographically intermediate coronary stenoses in patients with stable angina (Fihn et al., 2012; Kolh et al., 2014; Knuuti et al., 2020). However, uptake of FFR in coronary catheter laboratories worldwide has remained low. Potential reasons for the low adoption rate of coronary physiology despite demonstrated clinical benefit of its use may include time consumption to perform FFR measurements, tries, no availability of adenosine, patient-related discomfort, contraindications, or lack of reimbursement (Hannawi et al., 2014; Gotberg et al., 2017).

The underuse of invasive FFR in clinical practice has motivated research towards non-invasive prediction of FFR. Most early attempts for non-invasive FFR prediction relied on solving the incompressible 3D Navier–Stokes equations in segmented coronary arteries (Taylor et al., 2013; Raissi et al., 2019; Liu et al., 2021). Due to the need to solve the fully coronary model, the time cost of its calculation is very high. There are also some simple computational FFR models: reduced-order physics such as 1D blood flow or lumped parameter models (Itu et al., 2012; Blanco et al., 2018; Boileau et al., 2018), and (2) purely data-driven approaches (Hae et al., 2018; Zreik et al., 2018). Despite the fast computation time of this model, it is only included for stenotic vessels and ignores the entire coronary hemodynamic environment. Therefore, considering the fully hemodynamic environment of the coronary artery and improving the calculation speed are the development of non-invasive prediction of FFR.

In this study, a non-invasive quantification of fraction flow reserve based on steady-state geometric multiscale models (FFR_SS_) was developed. It was based on the coronary artery model segmented from the patient’s coronary CTA image. The average pressure was used as the boundary condition for the inlet, while the microcirculation resistance calculated by the coronary flow was used as the boundary condition for the outlet. Thus, it could rapidly calculate the patient-specific coronary hyperemia. Also, the flow velocity and pressure distribution were calculated, and the FFRss of each branch of the coronary artery was computed to evaluate the degree of myocardial ischemia caused by coronary stenosis. FFR_SS_ and FFR_CT_ were calculated simultaneously for 136 patients. The clinically measured FFR was used as the gold standard to verify the diagnostic performance of FFR_SS_, and the ability of FFR_SS_ and FFR_CT_ to evaluate myocardial ischemia was compared.

## Methods and Materials

### Patient and Image Data

**FIGURE 1:**
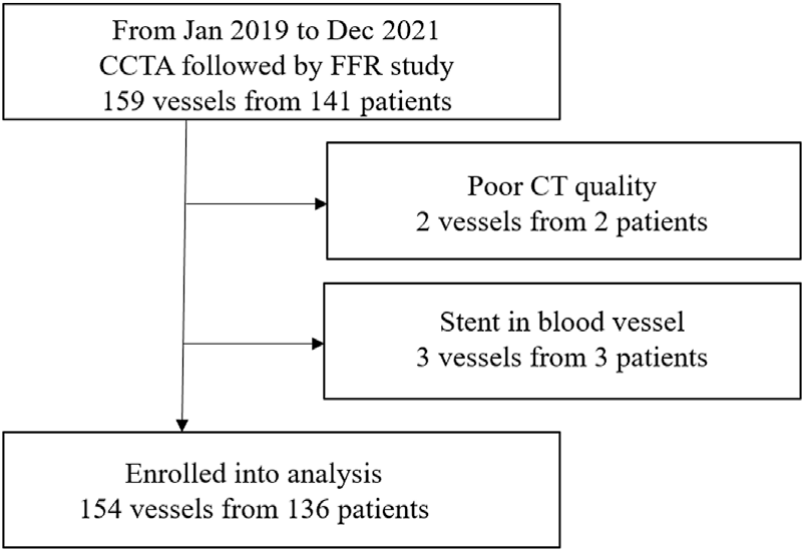
Study enrolment.

This study was approved by the institutional review board of Peking University People’s Hospital and the Second Affiliated Hospital of Zhejiang University School of Medicine. All patients signed an informed consent. 136 coronary heart disease patients with 154 moderate-to-severe epicardial stenosis were retrospectively enrolled (between 2019 and 2021). The patient inclusion criteria were shown in Figure 1. Under the guidance of coronary angiography based on the Azurion 7M20 DSA system, all patients had undergone the FFR catheter surgery measurement with FFR system and Verrata Plus pressure guide wire (Philips Healthcare, Netherland). The period between the CTA examination and the cardiac catheterisation did not exceed 1 week. The Biomechanics Laboratory of Beijing University of Technology analyzed the anonymized data independently.

The coronary CTA images were obtained using of a dual-layer detector CT system (IQon, Philips Healthcare), with a matrix size of 512 × 512 and a slice of 0.625 mm thickness. Segmentation and 3D reconstruction of the coronary artery for each patient were performed using Mimics (Materialise, Leuven, Belgium), with the results being reviewed by two radiologists with 15 years of experience in cardiac CTA. Only arteries with a diameter bigger than or equal to 1 mm were retained in the reconstructed model for further computational fluid dynamics (CFD) analysis (Sankaran et al., 2016).

### The Establishment of Steady-State Geometric Multiscale Models

In a previous study, we proposed a closed-loop geometric multiscale model to compute FFR (Liu et al., 2021) non-invasively. Although the closed-loop model has improved computational accuracy, the clinical application is limited due to its superior computational speed and complexity in determining individualized parameters. In order to fulfill the needs of clinical FFR calculation, a steady-state-based model mimicking the closed-loop geometric multiscale model was proposed in this study. It replaced the transient state with steady-state boundary conditions to reduce the computation time, optimize the geometric multiscale module, and reduce the optimization of individual parameters.

**FIGURE 2:**
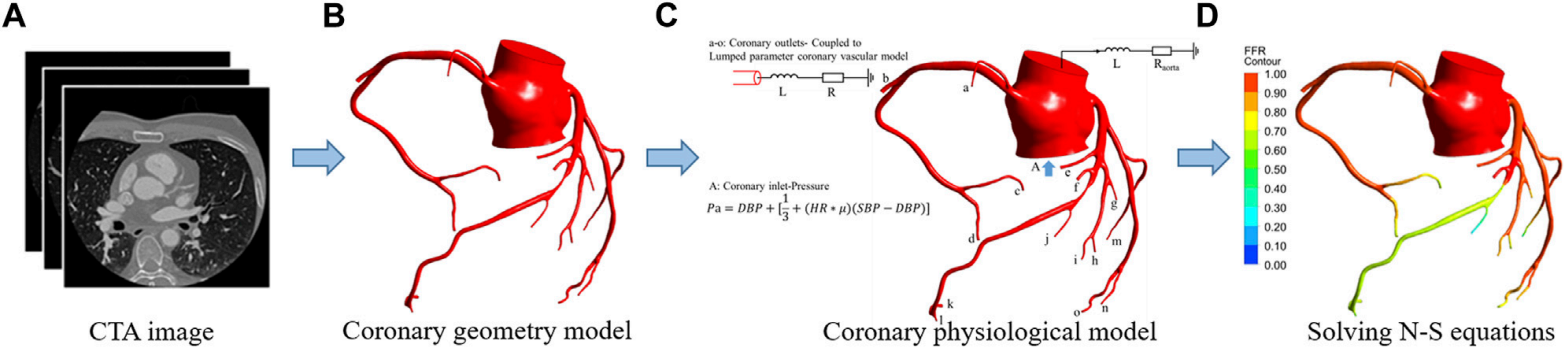
FFR_SS_ calculation flowchart. **(A)** CTA image. **(B)** Coronary geometry model. **(C)** Coronary physiological model. The 0D model stands for lumped parameter model (a–o) and is composed of resistance “R" and inductance “L.” “R” represents the flow resistance of coronary branch vessels under the action of blood viscosity, obtained by the allometric scale rate. “L” The inertia of blood flow, for the branch of the coronary artery, the empirical parameter is 0.05. The 3D model represents the whole coronary model, and the blood vessels larger than 1 mm in diameter are completely preserved. The 3D model was obtained by reconstruction of coronary CTA images. Among them, in the 0D-3D coupling model, the input is the mean pressure calculated by the physiological formula, and the output is the 0D resistance boundary condition of the coronary artery. **(D)** Solved N-S equations.0D and 3D coupling diagram.

The steady-state geometric multiscale model consists of lumped parameter model (LPM) (0D) and a coronary global three-dimensional (3D) model. LPM uses the circuit structure to simulate the microcirculation network downstream of the coronary artery, the resistance “R” to simulate the resistance of blood flow, and the inductance “L” to simulate the inertia of blood flow (Pietrabissa et al., 1996). The full-scale 3D model of the coronary artery preserves the real structure of the coronary artery instead of only stenotic vessels. The 0D part provides the outlet boundary conditions for the patient’s 3D coronary model. As shown in Figure 2C, the details of the geometrical multiscale model of 0D and 3D coupling are described previously (Zhao et al., 2016; Li et al., 2020; Mao et al., 2020).

The inlet boundary condition of the coronary model is set to the aortic pressure, which could be equivalent to the mean pressure (Wilson et al., 2001) and calculated from the cuff pressure based on systolic blood pressure (SBP), diastolic blood pressure (DBP), and heart rate (HR) (Sharma et al., 2012) as follows:

$$\text{Pa}=\text{DBP}+\left[\frac{1}{3}+\left(\text{HR}*0.0012\right)\left(\text{SBP}-\text{DBP}\right)\right]$$ (1)

The outlet boundary condition of the branch of the coronary model is composed of microcirculation resistance. The microcirculatory resistance of the downstream branch of the coronary artery was termed as resistance “R,” which could be estimated as follows:

$${\text{R}}_{\text{resting}}=\frac{\text{P}}{\text{Q}}$$ (2)

where P is the aorta pressure, and Q is the flow rate of blood in the target coronary branch while resting. The latter can be estimated using Murray’s Law (Murray, 1926) based on the patient’s cardiac output (Opie, 2003). Since the FFR needs to be calculated in the hyperemia state, according to the assumption (Wilson et al., 1990; Sdringola et al., 2011; Taylor et al., 2013), the R_resting becomes 0.24 of the original in the hyperemia state:

$${\text{R}}_{\text{hyperemia}}=24\%*{\text{R}}_{\text{resting}}$$ (3)

Notably, in the computation model of FFR_SS_ for the resistance of the outlet of the ascending aorta (Figure 2C) connected to the systemic circulation, the resistance is calculated based on the cardiac output (Opie, 2003):

$${\text{R}}_{\text{doa}}=\frac{Pa}{co}*96\%$$ (4)

where Pa is the pressure at the aorta pressure, and CO is the cardiac output.

### 0D/3D Interface Processing

**FIGURE 3:**
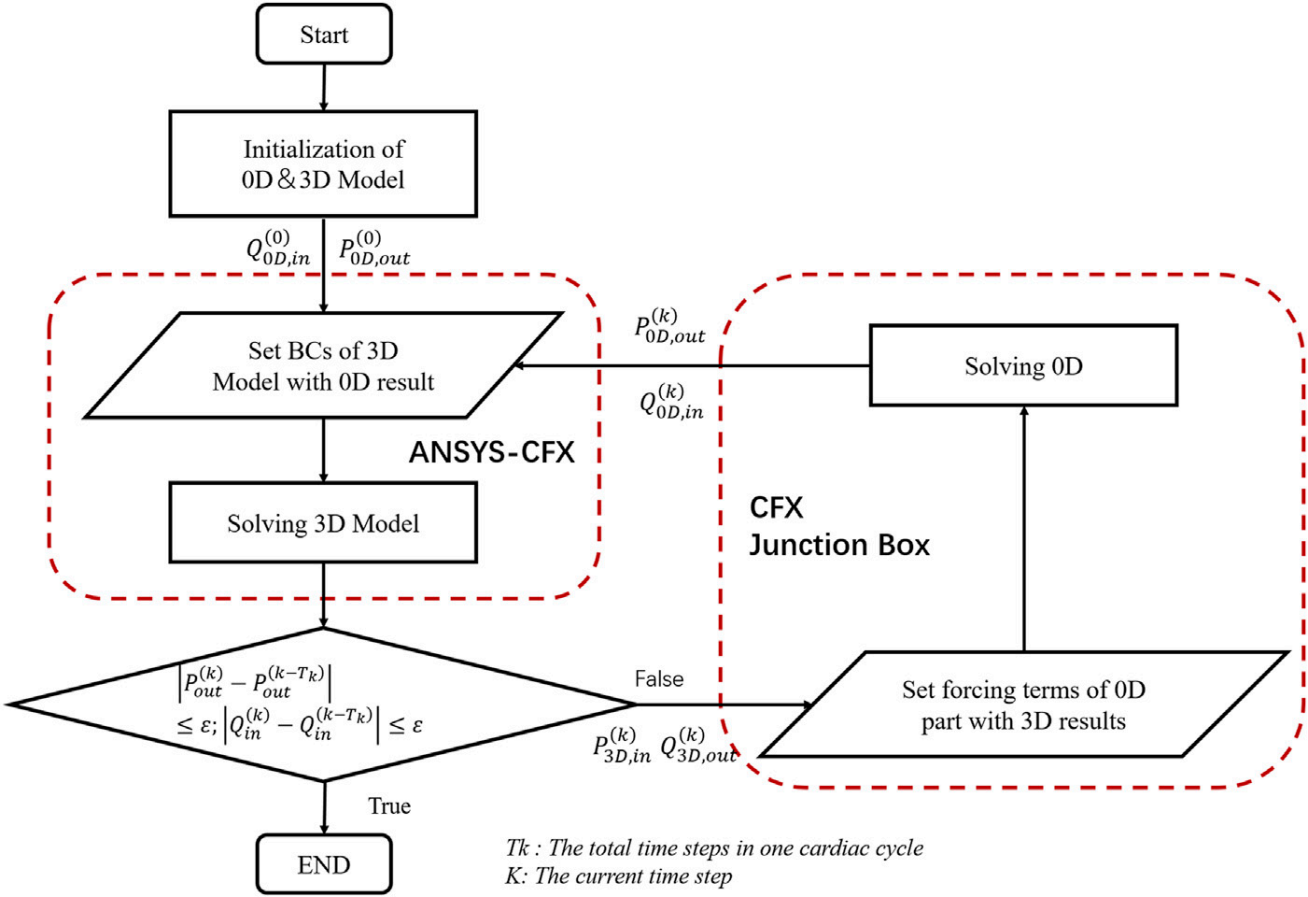
The solution flow chart of the geometric multiscale coupled model. where Q_(0D,in) represents the flow of the entrance at the junction of the 0D model and the 3D model. P_(0D,out) represents the average pressure at the outlet at the junction of the 0D model and the 3D model. P_(3D,in) represents the mean pressure at the inlet calculated by the 3D model. Q_(3D,out) represents the outlet flow calculated in 3D, and BC is short for boundary conditions. The formula in the figure is the coupling judgment formula of the 3D and 0D models, where ε = 0.0001.

The model described in this study was similar to the 0D/3D coupling method (Zhao et al., 2016; Liu et al., 2021) of the previous closed-loop model and used specific interface conditions and coupling algorithms to establish the 0D-3D coupling model. The 3D model calculation of the whole coronary artery relies on the fluid calculation software ANSYS, while the calculation of the lumped parameter model relies on the FORTRAN program of the CFX junction box. The data transmission between them was completed by the CFX User CEL Function, and the specific geometric multiscale coupling model solution process is shown in Figure 3.

The above models were divided into tetrahedral meshes by the ANSYS ICEM CFD software. It was assumed that the vascular wall was rigid and impermeable without slippage, the blood material property was adiabatic and comprised of an incompressible viscous Newtonian fluid, and its flow was unsteady laminar flow. The density of blood flow was set to 1,050 kg/m^3, and the viscosity of blood was set to 0.0035 Pa s (Deplano et al., 2001).

### Calculation Process of FFR

The specific steps of the steady-state geometric multiscale model include four processes: 1) Based on the coronary CTA image, a patient’s accurate personalized epicardial coronary 3D model was established; 2) According to the segment 3D model, the boundary conditions of the inlet (mean pressure) and outlet (microcirculation resistance) were calculated, respectively; 3) The change in the coronary microcirculation resistance in the maximum hyperemia state was quantified, and 0D and 3D coupling was calculated; 4) The Navier–Stokes (N-S) equation of the intracoronary fluid was solved using the ANSYS software, the flow velocity and pressure in each coronary artery were obtained under the hyperemia state, and the FFR_SS_ was calculated (Figure 2).

The specific steps of closed-loop geometric multi-scale calculation of FFR_CT_ include five processes: 1) Based on the patient’s coronary artery CTA image, the patient’s accurate and personalized 3D model of the epicardial coronary artery and heart model is constructed. 2) According to the constructed three-dimensional model and based on the allometric scaling law, the branch flow of the coronary arteries and the coronary microcirculation resistance in the resting state (assuming that there is no stenosis) are determined. 3) A closed-loop 0D centralized parameter model is constructed to personalize the physiological parameters of the patients. 4) The change in the coronary microcirculation resistance under maximum hyperemia is quantified, and zero-dimensional and three-dimensional coupling calculations are performed. 5) The control equation (N-S) of the fluid in the coronary artery is solved using a calculation software to obtain the flow velocity and pressure in each blood vessel of the coronary artery under congestion, and FFRCT is calculated. The detailed of FFR_CT_ calculation steps refer to previous studies (Liu et al., 2021).

In this study, we calculated the FFR of 134 cases based on the steady-state and closed-loop geometric multiscale model and compared the computational accuracy of FFR_SS_ with clinical FFR and FFR_CT_, respectively.

### Statistical Analysis

Data are summarized by descriptive statistics. Pearson correlation and linear regression analysis were performed to examine the relationship between FFR and FFR_SS_ and FFR_CT_, respectively. Agreement between the methods was assessed by Bland-Altman plots with corresponding 95% limits of agreement. The optimal cut-off values for FFR_SS_ was computed based on maximizing the sum of sensitivity plus specificity. The sensitivity, specificity, accuracy, and area under the ROC curves (AUC) with 95% CI classification metrics were computed. Throughout this study, a *p*-value threshold of 0.05 was considered to infer statistically significant findings.

## Results

### Characteristics of the Patients

A total of 154 vessels in 136 patients (57% male, median age:60 years) were analyzed with stenosis severity of coronary lesions evaluated by CCTA and ICA ranging from 40 to 80% luminal narrowing. Invasive FFR interrogation assessed the presence of hemodynamically significant stenosis (FFR ≤0.80) in 154 vessels (28.57%, 44/154) of 136 patients. The clinical and demographics characteristics of the patients’ population are summarized in Table 1.

**TABLE 1:** Basic characteristic form of enrolled patients.

Characteristic	Data
Number of patients	136
Number of vessels	154
Ages(years)	60 ± (10)
Male	78
Female	58
Systolic and diastolic blood pressure	128 ± (10)/85 ± (9)
Heart rate	72 ± (12.76) n/min
Cardiac output	5.26 ± 2.6 L/min
Myocardial mass	126 ± (34.08) g
Stenosis location	
Left artery descending (LAD)	115
Left circumflex artery (LCX)	11
Right coronary artery (RCA)	28

### Relationships Between FFR, FFR_SS_ and FFR_CT_

**FIGURE 4:**
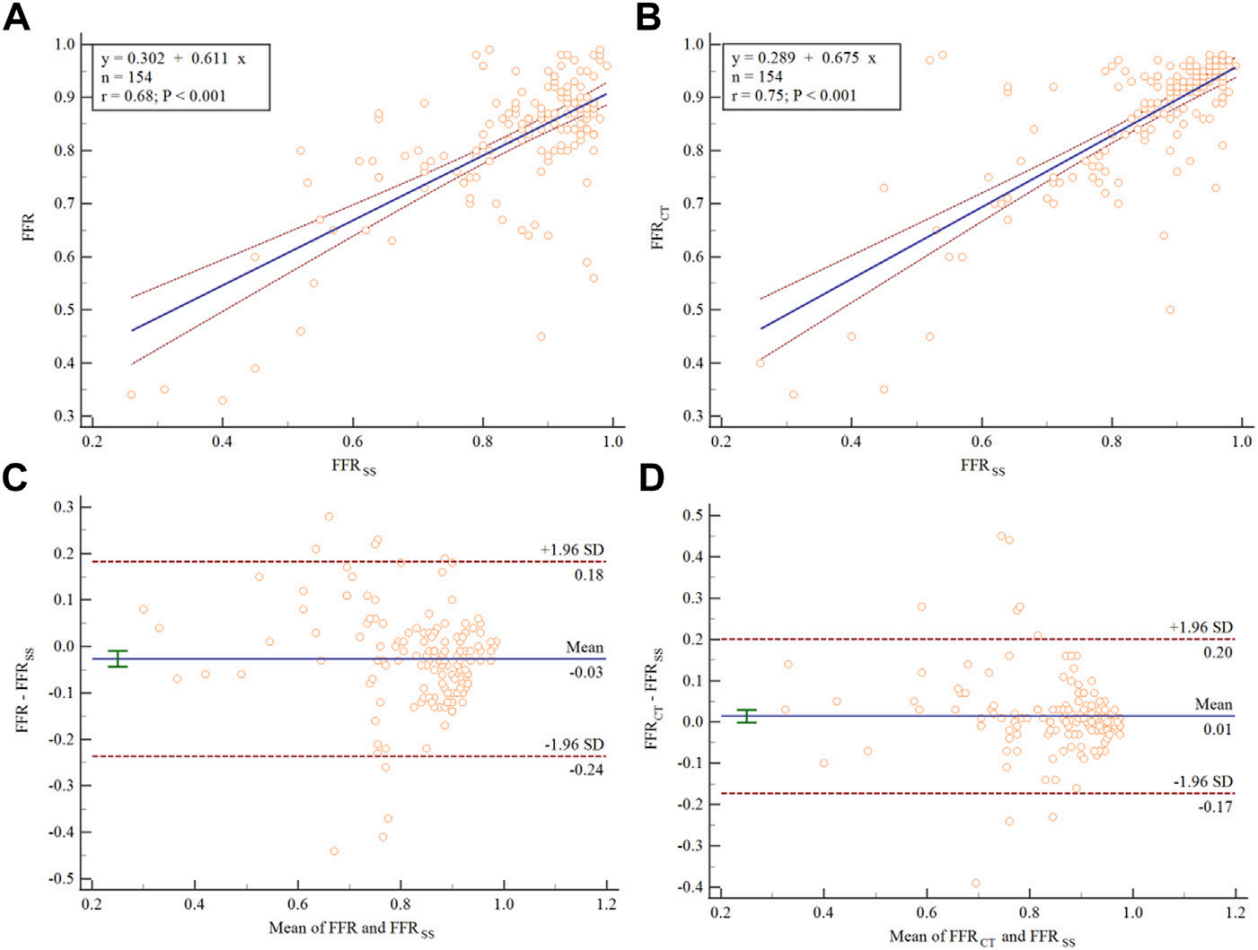
Scatter plot and Bland–Altman analysis showing the correlation between FFR, FFR_SS_, and FFR_CT_. The dashed blue line represents the line of best fit. **(A)** The correlation between FFR and FFR_SS_. **(B)** The correlation between FFR_CT_ and FFR_SS_. Bland–Altman plots of differences against the means are displayed for **(C)** FFR_SS_ and **(D)** FFR_CT_. The mean bias is represented by the solid blue line (with the 95% confidence interval represented by the dashed blue line).

The medians (interquartile range) of the FFR, FFR_CT_ and FFR_SS_ in this study were 0.81 (0.33–0.99), 0.85 (0.34–0.98), and 0.84 (0.26–0.99), respectively. A scatter plot between FFR and FFR_SS_ is shown in Figure 4A, demonstrating moderate overall linear correlation between the 2 measures, with an r of 0.68 (95% confidence interval [CI]:0.21–0.39) (*p* < 0.001). Similarly, the correlation of FFR_SS_ and FFR_CT_ demonstrated an r of 0.75 (95%CI: 0.67–0.72) (*p* < 0.001) (Figure 4B).

Bland-Altman plots for FFR_SS_ are illustrated in Figure 4C. On average, FFR_SS_ exceeded FFR by 0.03 (95% CI: −0.043 to −0.009). Most of the points in the figure are distributed within the 95% confidence interval, indicating that there is good agreement between FFR and FFR_SS_. Similarly, Bland-Altman plots for FFR_SS_ and FFR_CT_ are illustrated in Figure 4D. FR_CT_ exceeded FFR_SS_ by 0.01 (95% CI: −0.001 to −0.029).

The formula for calculating relative error:

$$\text{Relative}\;\text{error}=\frac{Calculate\;FFR-Clinical\;FFR}{Clinical\;FFR}$$ (5)

The relative error between FFR_SS_ and FFR is 0.11. The relative error between FFR_SS_ and FFR_CT_ is 0.067.

### Diagnostic Accuracy of FFR_SS_

The diagnostic performance of FFR_SS_ and FFR_CT_ are assessed using clinically measured invasive FFR as the diagnostic criteria. The Youden index was 0.61 and the optimal cut-off was 0.80 for FFR_SS_. The overall sensitivity, specificity, PPV, and NPV for FFR_SS_ ≤ 0.80 versus FFR ≤ 0.80 was 68.18% (95% CI: 52.4–81.4), 93.64% (95% CI: 87.3–97.4), 82.9%, and 91.1%, respectively, with an overall diagnostic accuracy of 88.3%. Similarly, the overall sensitivity, specificity, PPV, and NPV for FFR_CT_ ≤0.80 versus FFR ≤0.80 was 68.1% (95% CI: 52.4–81.4), 94.5% (95% CI: 88.5–98.0), 82.0%, and 89.5%, respectively, with an overall diagnostic accuracy of 87.6%.

**FIGURE 5:**
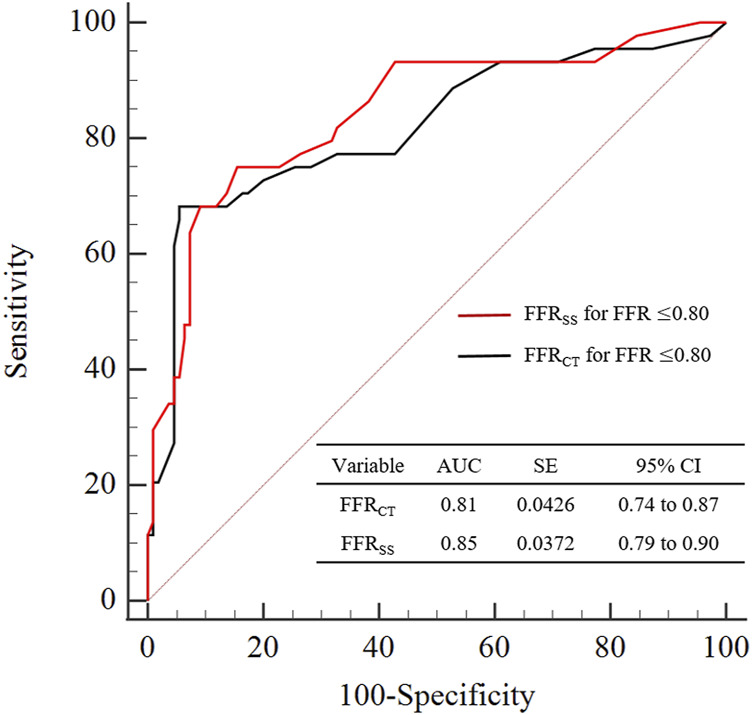
Graphs show diagnostic performance of FFR_SS_ and FFR_CT_. The diagnostic performance of FFR_SS_ and FFR_CT_ was compared with clinical FFR≤0.80 as the criterion for the diagnosis of myocardial ischemia. It can be seen from the figure that the diagnostic performance of FFR_SS_ and FFR_CT_ is comparable, both have good diagnostic performance, and the variance is also close. AUC = area under receiver operating characteristics curve.

According to the ROC receiver characteristic curve, the area under the curve of FFR_SS_ and FFR_CT_ are AUC = 85.7% (95%CI: [0.79–0.90]), AUC = 81.8% (95%CI: [0.74–0.87]), respectively. It suggesting that a good diagnostic performance is achieved of FFR_SS_ as shown in Figure 5.

### Hemodynamic Results of Coronary Artery Stenosis

**FIGURE 6:**
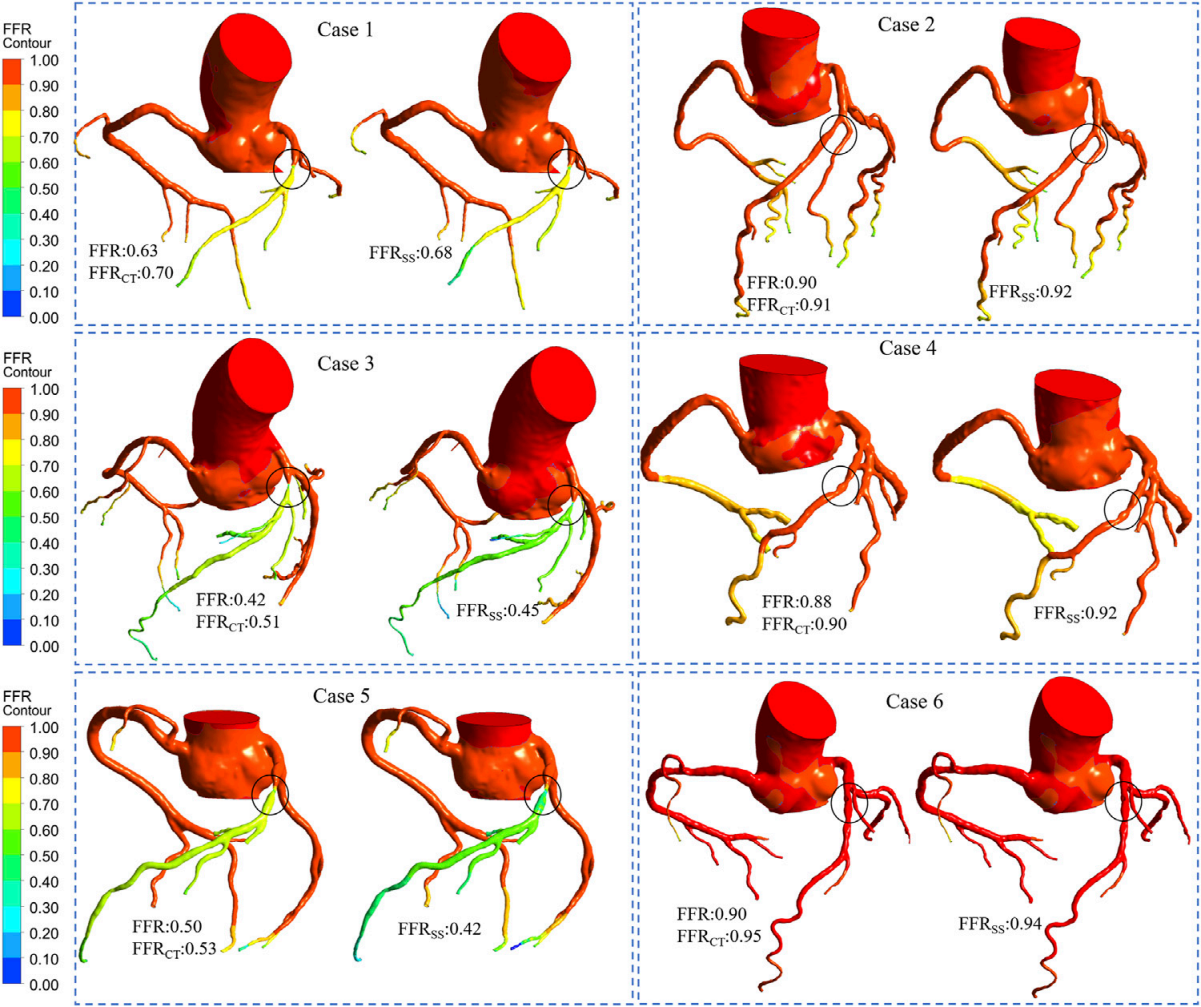
Computed FFR contours for representative patients. Six representative patients had moderate stenosis of the left descending artery. Among them, cases 1, 3, and 5 were ischemia patients. Cases 2, 4, and 6 were non-ischemic patients. The FFR, FFR_CT_, and FFR_SS_ for each representative patient are listed separately. It can be seen from the figure that the contours of the calculation result of FFR_CT_ is comparable to that of FFR_SS_, and it have good consistency.

The FFR_SS_ analysis of 6 representative patients was based on each narrowed vessel. We also list the clinical FFR, FFR_CT_ and FFR_SS_ of representative patients. The hemodynamic differences between FFR_CT_ and FFR_SS_ were compared based on clinically measured FFR (Figure 6). The stenosis of 6 representative patients stenosis was located in the anterior descending artery (LAD), and the degree of stenosis was 40–80%. It can be seen from the figure that the hemodynamic distribution calculated by FFR_SS_ is basically consistent with that of FFR_CT_.

## Discussion

A rapid method for calculating FFR is proposed in this study. Based on the closed-loop geometric multiscale model for calculating FFR, the transient pressure boundary condition at the inlet was changed to a steady-state, and the model was optimized to ensure calculation accuracy. The inlet boundary condition improves computational efficiency. The diagnostic performance of FFR_SS_ was validated by clinical FFR of 136 personalized patients. The FFR_CT_ was calculated at the same time as the FFR_SS_, and the myocardial ischemia assessment ability of the two calculated FFR methods was compared. The computational results showed that FFR_SS_ was correlated and in agreement with both FFR and FFR_CT_, with excellent diagnostic performance.

### Advantages of FFR_SS_ Compared to FFR_CT_

**FIGURE 7:**
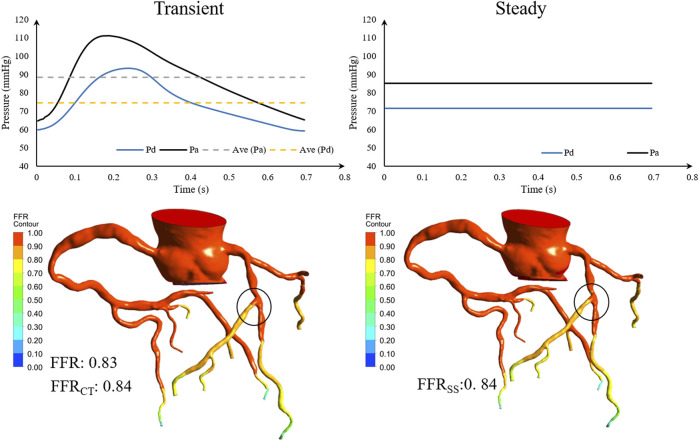
Geometric multiscale solution for comparison of transient and steady-state pressure. Pa is the inlet pressure of the stenotic vessel, Pd is the outlet pressure, and Ave is the mean pressure.

In the previous closed-loop geometric multiscale model (Liu et al., 2021), the physiological parameters of the patient had to be optimized to simulate the individualized physiological state, using transient periodic inlet boundary conditions. The FFR_SS_ model adjusts the transient boundary condition of the inlet to a steady-state and replaces the transient pressure with the average pressure, which markedly improves the calculation efficiency. Then, the transient and steady-state pressure waveforms of the coronary arteries calculated by the geometric multiscale model were compared (Figure 7). FFR_SS_ can replace FFR_CT_ because the steady-state pressure and the transient average pressure are the same, resulting in the same calculation of FFR.

In addition, the FFR_SS_ model saves the tedious process of optimizing the cardiac parameters of individual patients and replaces it with the average pressure, thereby improving the calculation efficiency and the clinical applicability of the model. The calculation time of FFR_CT_ is usually 8–9 h, while the calculation time for FFR_SS_ is only 20 min. Unlike other models that only consider stenotic vessels to calculate FFR (Itu et al., 2012; Itu et al., 2016; Zreik et al., 2018), the calculation model described in this study retains the complete coronary model, which can view the complete hemodynamic state of the coronary artery.

### The Selection of Inlet and Outlet Boundary Conditions

The inlet of the FFR_SS_ model adopts the mean pressure calculated based on the “physiological formula,” and the outlet adopts the microcirculation resistance model as the closed-loop geometric multiscale model. In a previous study, we presented a numerical investigation of the effects of the computational model’s inlet and outlet boundary conditions on computed CT-FFR. The mean pressure calculated by the “physiological formula” differed from the real aortic pressure wave (Tosello et al., 2021). However, the calculation model was not sensitive to the boundary conditions of the inlet pressure, i.e., the true aortic pressure could be replaced by the mean pressure calculated by the “physiological formula.” The findings revealed that distal boundary conditions (hyperemic vasodilation response of coronary micro-vessels) have a significant impact on FFR. Thus, improving the calculation accuracy of distal microcirculation resistance is the key to further improving the calculation of FFR_SS_.

### Diagnostic Performance of FFR_SS_

The calculation results show that compared to the closed-loop geometric multiscale model, the improved calculation model does not reduce the accuracy of calculating FFR. The accuracy of traditional FFR_CT_ is 84.3% (Taylor et al., 2013), and that of FFR_CT_ based on the closed-loop geometric multiscale model is 87.3% (Liu et al., 2021), which was similar to the 88.3% computational accuracy of FFR_SS_ proposed in this study. Compared to the closed-loop geometric multiscale model, the FFR_SS_ improves the computation speed, retaining the computation time within half an hour. Itu et al. proposed a non-invasive FFR calculation based on a neural network with an accuracy rate of 88.3% (Itu et al., 2016). Fredrik et al. (Fossan et al., 2021) proposed a non-invasive rapid calculation method of FFR based on an enhanced neural network, while the standard deviation of repeated FFR measurements was 0.018. However, the premise of improving the calculation speed in the above two studies was that only the coronary arteries in the stenotic segment are retained, and the other coronary arteries are ignored. The advantage of this study is that while improving the calculation speed, it retains the complete model and displays the hemodynamic positions of all coronary arteries, facilitating the diagnosis of myocardial ischemia.

## Limitations

CT-based non-invasive FFR_SS_ calculations are very sensitive to image quality and segmentation models. FFR_SS_ requires accurate anatomical models. Image artifacts, calcifications and improper registration may limit the accuracy of model calculations. Therefore, it is important to follow the protocol of high-quality image data and accurate description of the boundary of the lumen (Zarins et al., 2013).

Although the FFR_SS_ computation model shortens the calculation time to less than half an hour, there is still some gap compared to the other simplified non-invasive methods for calculating FFR (Itu et al., 2016; Fossan et al., 2021), which does not meet the requirements of real-time FFR calculation. In the future studies, we will directly predict the coronary flow field through a neural network based on that calculated by the steady-state model FFR_SS_, thereby improving the calculation speed.

## Conclusion

The present study proposed a steady-state-based geometric multiscale model to calculate FFR non-invasively and validate its accuracy with personalized clinical data from 136 cases. The calculation method has the same accuracy as the closed-loop geometric multiscale FFR computation but reduces the calculation time and exhibits a satisfactory diagnostic performance. This method improves the clinical applicability of the non-invasive computational FFR model, helps clinicians diagnose myocardial ischemia, and guides percutaneous coronary intervention (PCI) operations.

## Data Availability

The raw data supporting the conclusion of this article will be made available by the authors, without undue reservation.
